# Mechanisms Underlying Improvement in Obstructive Sleep Apnea Syndrome by Uvulopalatopharyngoplasty

**DOI:** 10.1155/2017/2120165

**Published:** 2017-06-06

**Authors:** Takahisa Yamamoto, Naoko Fujii, Yoichi Nishimura, Noboru Iwata, Seiichi Nakata

**Affiliations:** ^1^Department of Mechanical Engineering, National Institute of Technology, Gifu College, Motosu, Japan; ^2^Department of Radiology, Second Hospital, Fujita Health University School of Medicine, Nagoya, Japan; ^3^Department of Otorhinolaryngology, Second Hospital, Fujita Health University School of Medicine, Nagoya, Japan

## Abstract

In a previous case report, we determined for the first time that uvulopalatopharyngoplasty (UPPP) does not change the volume of the upper airway but causes morphological changes in the entire upper airway. The objective of this study is to elucidate the mechanisms underlying the improvement in obstructive sleep apnea syndrome (OSAS) by UPPP. We present an additional case involving a patient with OSAS treated using UPPP. Morphological and numerical parameter changes after surgery were compared with the corresponding preoperative values. Anatomically accurate upper airway computational models were reconstructed from computed tomographic imaging data. In addition, computed fluid dynamics analysis was performed to reveal inhalation flow characteristics before and after UPPP and clearly assess the effect of UPPP on airflow patterns in the patient's upper airway. An important benefit of UPPP is the morphological changes in the entire upper airway, in addition to widening the restricted area. These morphological changes induce laminarization of the pharyngeal jet. To obtain sufficient efficacy of UPPP in OSAS, the morphological changes in the upper airway and the airflow pattern after the surgery must be controlled.

## 1. Introduction

Uvulopalatopharyngoplasty (UPPP) is one of the standard conventional techniques for the treatment of obstructive sleep apnea syndrome (OSAS); it is performed in cases with severe OSAS. In UPPP, excess tissue in the throat is removed to widen the patient's airway. This procedure may facilitate air movement through the throat more easily when the patient breathes, thereby reducing the severity of OSAS. Some mechanisms by which UPPP improves OSAS have been suggested. However, these mechanisms remain controversial even though UPPP is a commonly performed procedure for serious cases of OSAS. Based on measured respiratory parameters, the success rate in long-term follow-up has been reported as only approximately 50% [1]. Recently, computational fluid dynamics (CFD) analysis has been used to characterize the fluid flow in human airway models [2–4]. CFD is of significant interest in both engineering and medical fields because of its noninvasive nature. It enables health professionals to predict fluid flow characteristics with a high definition when the fluid flow conditions, such as the mean inspiratory/expiratory flow rate, respiratory rate, input flow turbulence, and lung pressure, are variable. Previously, we had performed morphological analysis in OSAS patients before and after UPPP; it revealed that the upper airway volume hardly changed pre- and postoperatively, although 15 g of bilateral tonsil mass was removed [5]. This study used the latest improvements in CFD analysis in conjunction with upper airway scans to characterize the upper airway response to UPPP, using both morphological and numerical parameters. An OSAS patient was selected after reviewing the varied treatment responses of our treated patients, as assessed using the apnea-hypopnea index (AHI) and overnight polysomnography (PSG). Anatomically accurate patient-specific upper airway models were reconstructed from computed tomography (CT) imaging data before and after UPPP. CFD analysis revealed inhalation flow characteristics before and after UPPP and clearly demonstrated the mechanisms and efficacy of UPPP.

## 2. Case Presentation

**Figure 1:**
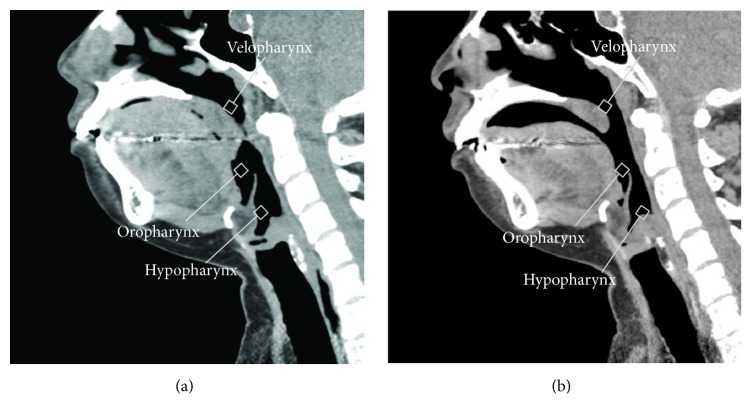
Mid-sagittal CT sections of the pharynx in patient 1 (a) before UPPP and (b) after UPPP.

The patient was a 36-year-old woman who had been treated with nasal continuous positive airway pressure (n-CPAP) for severe OSAS, with an AHI of 112.1/h, a desaturation rate (DR) of 45.0%, and a lowest oxygen saturation (LSAT) of 61.0%. Because she was intolerant of n-CPAP and had large tonsils, we performed UPPP under general anesthesia. Her bilateral palatine tonsils, one-third of the uvula, and the anterior and posterior pillars were removed. The weights of the removed right and left tonsils were 7.6 g and 7.5 g, respectively. Postoperative full-night PSG was performed 2 months after UPPP. The PSG results showed an AHI of 3.8/h, a DR of 0.2%, and an LSAT of 84.0%. The patient's Epworth sleepiness scale score had improved to 6 from a previous score of 17 before UPPP, thereby showing a marked improvement in OSAS grade. Nevertheless, we could easily elucidate the mechanism and efficacy of UPPP in this case. A three-dimensional, anatomically accurate, patient-specific model was reconstructed from the data obtained using a 64-row multi-detector CT scanner (Brilliance 64; Philips Medical Systems, Cleveland, OH, USA) with a medical imaging software package (Mimics; Materialise, Leuven, Belgium). The entire series was loaded into the software, and then the upper airway was identified in each of the axial images based on a predefined threshold of 250 Hounsfield units relative to the surrounding tissue.  Figure 1 shows mid-sagittal sections before (Figure 1(a)) and after (Figure 1(b)) UPPP in the patient.

### 2.1. Morphological Changes in the Upper Airway

**Figure 2:**
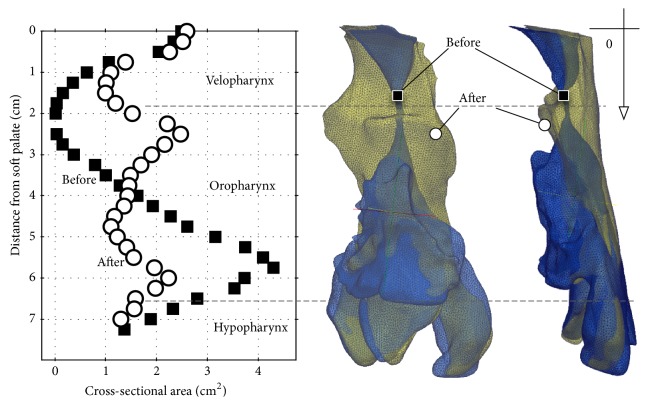
Three-dimensional model of the upper airway and cross-sectional area data alongside the airway in patient 1. The morphological characteristics of the upper airway are markedly changed after UPPP. However, there is little change in the upper airway volume after UPPP (11.28 cm^3^ before and 11.78 cm^3^ after UPPP).

Figure 2 shows the cross-sectional area along with the upper airway from the upper end of the velopharynx to the hypopharynx. The morphological characteristics of the upper airway changed markedly before and after UPPP. However, the volume measurements before (11.28 cm^3^) and after (11.78 cm^3^) UPPP showed negligible change. The difference in cross-sectional area before UPPP treatment relative to that after UPPP treatment was significant. The minimum and maximum areas before UPPP were 0.15 cm^2^ and 2.5 cm^2^, respectively; in contrast, the minimum and maximum areas after UPPP were 1.0 cm^2^ and 2.6 cm^2^, respectively.

### 2.2. Inhalation Flow Analysis

The upper airway model constructed using the Mimics software package was exported into the ANSYS ICEM CFD meshing software package (ANSYS 15.0; ANSYS, Canonsburg, PA, USA) to generate discrete volume cells. Unstructured tetrahedral volume meshes were generated in the airway surface model. The upper airway geometry had an irregular shape, with bends and changes in areas, that showed features such as laminar-to-turbulent transitions, adverse pressure gradients, secondary flow regions, and recirculation zones. This study used a* k*-omega-based shear stress transport model, which is an advanced* k*-omega model [6, 7] that is able to account for the transport of the turbulent shear stress and to consequently and accurately predict the flow separation amounts under adverse pressure gradients. In the current study, we assumed that the flow was uncompressible. CFD analysis was performed for an inspiratory volumetric flow rate of 6 L/min. The turbulence intensity was set to 10% to mimic real conditions, and an average gage pressure of 0 Pa was defined at the outlet.

**Figure 3:**
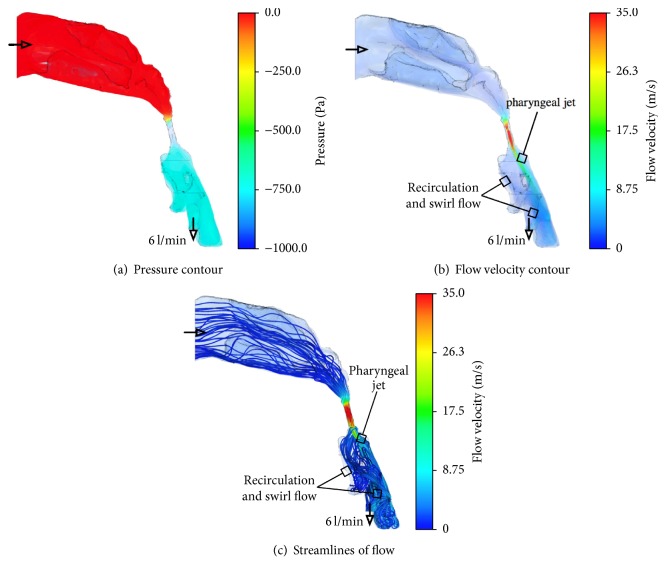
Streamlines and contour maps of pressure and velocity before UPPP; (a) pressure contour, (b) flow velocity contour, and (c) streamlines. Low-pressure regions are located at the distal velopharynx, oropharynx, and hypopharynx. At the velopharynx, a high velocity indicates a low static pressure. Vertical recirculation and swirl flow is observed where the airway narrows around the epiglottis between the oropharynx and hypopharynx.

**Figure 4:**
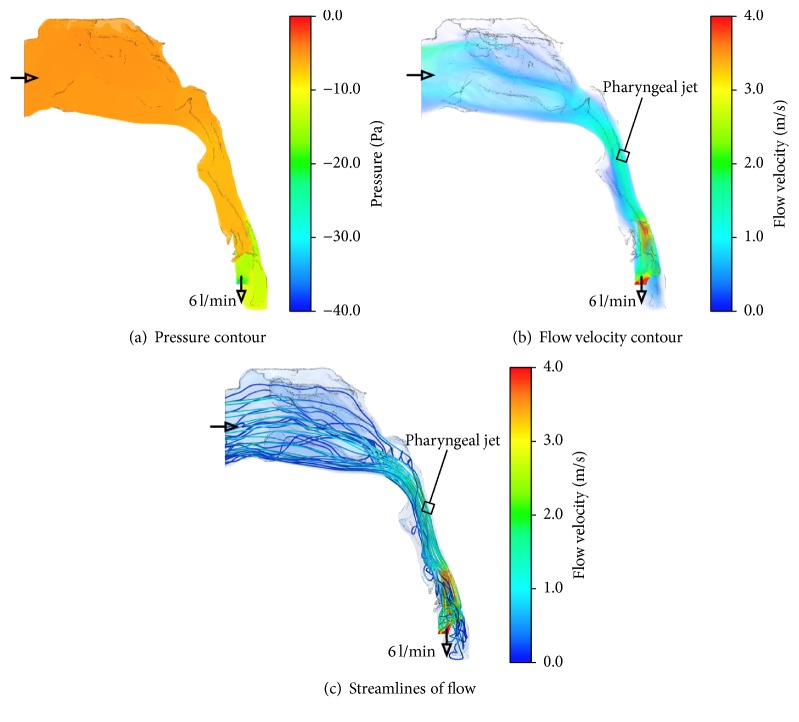
Streamlines and contour maps of pressure and velocity after UPPP; (a) pressure contour, (b) flow velocity contour, and (c) streamlines. The complex flow observed before UPPP is inhibited and laminarized after UPPP.

Figures 3 and 4 show the inhalation flow characteristics and the streamlines and contour maps of the flow velocity and pressure, respectively, before and after UPPP. The pressure contours of the upper airway before UPPP indicated that low-pressure regions were located at the distal velopharynx, oropharynx, and hypopharynx. At the velopharynx, a high velocity indicated a low static pressure, as per Bernoulli's theorem. As shown in the streamlines, the airflow initially converged behind the uvula and then formed a jet flow near the posterior wall of the oropharynx and hypopharynx before traveling through the vocal cords. Before UPPP, vertical recirculation and swirl flow was observed where the airway narrowed around the epiglottis between the oropharynx and hypopharynx. In contrast, this complex flow was inhibited and laminarized after UPPP.

## 3. Discussion

This study showed for the first time that UPPP can cause morphological changes in the entire upper airway, without any changes in air volume. Furthermore, these morphological changes inhibited the pressure gradient and laminarized the complex recirculation flow in the upper airway.

Previous studies on OSAS and UPPP have mainly focused on widening the most restricted region in the upper airway. In an upper airway analysis using magnetic resonance imaging in a patient with OSAS, Langin et al. demonstrated that the upper airway was restricted along the upper two-thirds of its length and that treatment effectively widened the restricted region [8]. In a comparative study using CT in patients with OSAS before and after UPPP, Heenan et al. demonstrated a widening of the fauces after treatment [9]. However, widening of the restricted region is not necessarily an important factor in OSAS treatment, because the success rate based on the measurement of respiratory parameters has been shown to be approximately 50% in long-term follow-up [1]. The morphological changes in the upper airway shown in Figure 2 indicate that the important benefits of UPPP are widening of the most restricted region at the velopharynx and the morphological changes in the entire upper airway after UPPP. Figures 3 and 4 show that the pressure gradient between the velopharynx and oropharynx was relatively large. This feature caused a jet flow and a large recirculation zone occurred in this region. The pressure profile was similar before and after the treatment, and the lowest pressure was at the velopharynx. Regarding the velocity contour results before treatment, a recirculation and swirl flow, known as the pharyngeal jet [10], occurred below the most restricted region of the airway at the velopharynx. The reason this recirculation and swirl flow forms in the velopharynx and oropharynx is because of flow separation between the pharyngeal jet and flow patterns, which then reverse and travel upstream and create a vertical recirculation flow within these regions. In contrast, no recirculation and swirl flow was observed at the oropharynx after treatment in our patient. The pharyngeal jet velocity had decreased relative to that before treatment because of the widened area of the velopharynx. Both a significant inward pressure force and an intensive recirculation and swirl flow can be generated from the pressure difference between a low pharyngeal pressure and the external tissue pressure on the airway wall, which can effectively collapse the airway. Our CFD analysis results showed that the benefits of UPPP are inhibition of both the pressure gradient and complex recirculation flow, which consequently laminarizes the pharyngeal jet flow. This mechanism is an important factor in the UPPP success rate in OSAS.

## 4. Conclusion

In this study, one of the mechanisms by which UPPP improves OSAS, both morphologically and numerically, was discussed. From the morphological viewpoint, the benefits are achieved by widening the most restricted region at the velopharynx and by morphological changes in the entire upper airway. Our CFD analysis showed that the effectiveness of UPPP stems from the inhibition of the pressure gradient and pharyngeal jet and from the laminarization of the complex recirculation flow in the upper airway. The results suggested that both the morphological changes in the upper airway and the pharyngeal flow patterns must be considered and controlled to obtain sufficient efficacy of UPPP in OSAS. Further studies using the techniques stated in this paper can estimate the efficacy of UPPP before the surgery.
